# Development and repair of blood vessels in the zebrafish spinal cord

**DOI:** 10.1098/rsob.230103

**Published:** 2023-08-09

**Authors:** Ana Ribeiro, Mariana Rebocho da Costa, Carmen de Sena-Tomás, Elsa Charas Rodrigues, Raquel Quitéria, Tiago Maçarico, Susana Constantino Rosa Santos, Leonor Saúde

**Affiliations:** ^1^ Instituto de Medicina Molecular—João Lobo Antunes, Faculdade de Medicina da Universidade de Lisboa, Lisboa 1649-028 Portugal; ^2^ Instituto de Histologia e Biologia do Desenvolvimento, Faculdade de Medicina da Universidade de Lisboa, Lisboa 1649-028 Portugal; ^3^ Centro Cardiovascular da Universidade de Lisboa (CCUL@RISE), Faculdade de Medicina da Universidade de Lisboa, Lisboa 1649-028 Portugal

**Keywords:** spinal cord, vascular development, vascular repair, zebrafish

## Abstract

The vascular system is inefficiently repaired after spinal cord injury (SCI) in mammals, resulting in secondary tissue damage and immune deregulation that contribute to the limited functional recovery. Unlike mammals, zebrafish can repair the spinal cord (SC) and restore motility, but the vascular response to injury has not been investigated. Here, we describe the zebrafish SC blood vasculature, starting in development with the initial vessel ingression in a body size-dependent manner, the acquisition of perivascular support and the establishment of ventral to dorsal blood circulation. The vascular organization grows in complexity and displays multiple barrier specializations in adulthood. After injury, vessels rapidly regrow into the lesion, preceding the glial bridge and axons. Vascular repair involves an early burst of angiogenesis that creates dysmorphic and leaky vessels. Dysfunctional vessels are later removed, as pericytes are recruited and the blood–SC barrier is re-established. This study demonstrates that zebrafish can successfully re-vascularize the spinal tissue, reinforcing the value of this organism as a regenerative model for SCI.

## Graphical abstract

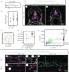

## 1. Introduction

Traumatic spinal cord injury (SCI) causes neuronal and glial loss and severs axonal tracts and blood vessels, which impact spinal cord (SC) function in the transmission of motor and sensory signals [1]. The severity of motor deficits depends on the strength and level of the injury and patient welfare may be worsened by systemic complications downstream of SC dysfunction. Although some neurological recovery may be observed, the restoration of motor function is generally limited in most patients and efficient therapies are still lacking or in experimental stages [2]. Studies in rodents have shown that the impact of SCI on the vascular system has both immediate and secondary consequences that worsen the injury outcome. The initial disruption of the vasculature leads to haemorrhage that causes further cell death and allows the extravasation of immune cells [3,4]. The blood-spinal cord barrier (BSCB) is disrupted within minutes of the injury [5] and can remain compromised for up to 10 weeks [6]. After SCI the vascular system is not correctly repaired, resulting in further leakage and ischaemia. Although some blood vessels grow into the lesioned tissue, these vessels have a compromised BSCB due to the disruption of tight junctions [7] and incomplete coverage by astrocytes [8,9]. As new blood vessels enter the lesion site, pericytes also detach from endothelial cells (ECs) and form scar tissue [10]. The immature vessels continue to leak toxic molecules and inflammatory cells into the tissue, thus contributing to the secondary damage [7,11,12]. The impaired vascularization in the damaged tissue has also been associated with cavitation [13].

The limited repair of the mammalian SC contrasts with other vertebrate models, such as zebrafish. Adult zebrafish are able to fully restore their motor function in four to six weeks after SCI [14,15]. The mechanisms underlying the successful regenerative program in zebrafish include the quick resolution of inflammation and absence of a glial scar [15]; the formation of new neurons that replace lost cells [16]; and axonal regeneration across the injury site and reconnection to targets [17]. However, little is known about the SC vasculature in adult zebrafish and how blood vessels respond to SCI. Previous studies in adult zebrafish show blood vessels in the proximity of the central canal [18–20] with an enrichment in the grey matter [21], and increased vessel density after SCI [19].

Here, we carried out a comprehensive analysis of blood vessel distribution and morphology during zebrafish SC development, homeostasis and regeneration. The organization and permeability properties of the spinal vascular network were disrupted by SCI. But unlike mammals, the zebrafish SC was able to re-establish a stable and functional vascular network, driven by endothelial proliferation, pericyte recruitment and BSCB recovery.

## 2. Results

### 2.1. Spinal cord vascularization in developing zebrafish

**Figure 1.**
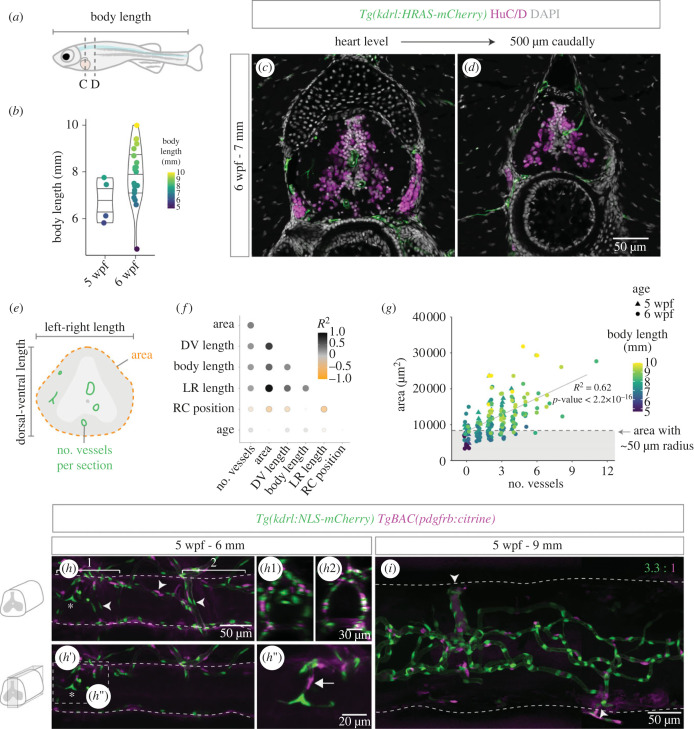
Spinal cord vascularization in juvenile zebrafish. (*a*) Definition of body length and rostral–caudal region analysed in juvenile zebrafish. (*b*) Body length (*y*-axis and symbol colour) in juvenile fish with 5 (*n* = 4) or 6 (*n* = 20) weeks post fertilization (wpf). (*c,d*) 6 wpf fish sections at the heart level (*c*) and 500 µm caudally (*d*) with labelled endothelial cells (*Tg(kdrl:HRAS-mCherry)*), HuC/D^+^ neurons and nuclear DAPI staining. (*e*) Schematic showing the parameters quantified in the imaged sections. (*f*) Correlation matrix dot plot of the quantified parameters: area; DV (dorsal–ventral) length; body length; LR (left-right) length; RC (rostral–caudal) position (starting at the heart level); age and number of vessels per section. Dot size and colour represent the Spearman correlation coefficient. (*g*) SC area relative to number of vessels per section in all sections analysed. Symbol shape indicates age and symbol colour indicates body length. Dashed line corresponds to average SC area in sections with 1 vessel inside. (*j*) 5 wpf juvenile with 6 mm with labelled endothelial nuclei (*Tg(kdrl:NLS-mCherry)*) and perivascular cells (*TgBAC(pdgfrb:citrine)*) (arrowheads identify the perineural vascular plexus and the asterisk marks a vessel inside the spinal cord). (*h*1,*h*2) Transversal views of the regions marked with brackets in *h*. (*h*′) Ingressing blood vessel in the central region with co-recruited mural cells (arrow) (magnification in *h*″). (*i*) A 9 mm fish SC with a more complex vasculature and high perivascular coverage (3.3 ECs per perivascular cell ±0.28, *n* = 3). Arrowheads label points of connection between the intraspinal network and external vessels.

The intraspinal tissue is vascularized only late in development, as blood vessels grow from the perineural vascular plexus (PNVP) that surrounds the SC and enter the neural tissue [20]. This process was described to occur between 12–14 [22] or 17–18 days post fertilization [20], suggesting that the timing of the entry cannot be predicted by age alone. Since body length can vary between fish with the same age (figure 1*a,b*), we asked if other parameters were better correlated than age with the entry of vessels into the SC. For this, we performed a correlation analysis between the number of vessels (*Tg(kdrl:HRAS-mCherry)*) and SC area, SC left–right length, SC dorsal–ventral length, rostral–caudal position and age, using trunk sections of five to six weeks post fertilization (wpf) fish to quantify these parameters (figure 1*c–e*). This analysis showed that the number of vessels had a high positive correlation with size-related parameters and low correlation with age (figure 1*f*), suggesting that the size of the fish and, in association, SC size, had the strongest influence in the timing of intraspinal vascularization. Likewise, the number of vessels was negatively correlated with rostral–caudal position, consistent with a rostral to caudal ingression. The average SC area in which vessels started to be detected inside (8378 ± 2106 µm^2^; figure 1*g*) corresponded to an approximate radius of 50 µm, suggesting that below this radius the PNVP is sufficient to perfuse the intraspinal tissue. At this stage, most cells in the SC are differentiated neurons (HuC/D^+^) (figure 1*c,d*), whereas in mouse and chicken vessels enter a more immature SC [23]. These data describe the size-dependent vascularization of the zebrafish SC at a later developmental stage than in mammals.

The mature SC microvasculature is coated by perivascular cells [24], including in zebrafish [25]. To determine if perivascular cells also accompany ECs as they enter the SC, we analysed 5 wpf fish with labelled mural cells/pericytes (*TgBAC(pdgfrb:citrine)*) and ECs (*Tg(kdrl:NLS-mCherry)*). We observed *pdgfrb*^+^ cells being co-recruited with the angiogenic sprouts as ECs invade the nervous tissue (figure 1*h–h*″), and a higher mural coverage (1 *pdgfrb*^+^ cell to 3.3 ECs) in the more developed vascular network (figure 1*i*). The vessels around the SC also have associated *pdgfrb*^+^ cells (figure 1*h*) [25], raising the possibility that PNVP mural cells are the source of intraspinal *pdgfrb*^+^ cells. These results show the coordinated entry and dispersal of ECs and perivascular cells during the formation of the SC vasculature.

**Figure 2.**
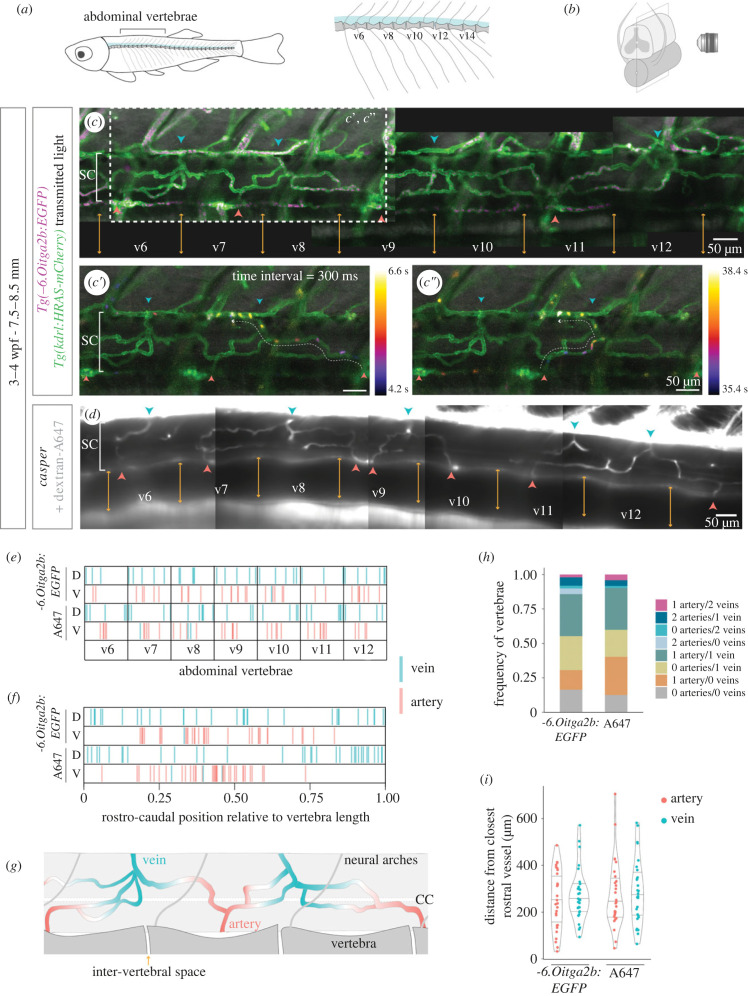
Direction of blood flow and distribution of arteries and veins in the developing spinal cord vasculature. (*a*) Schematic of vertebral column in zebrafish, with a magnified view of abdominal vertebrae on the right. (*b*) Schematic of the SC plane acquired during live imaging. (*c*) Projection of 500 time-frames (300 ms interval) acquired in live 3–4 wpf fish with labelled ECs (*Tg(kdrl:HRAS-mCherry)*) and thrombocytes (*Tg(-6.0itga2b:EGFP)*) (*n* = 7). The intervertebral spaces are labelled with orange arrows, arteries in red and veins in blue. *c*′,*c*″. Projections of the region shown in (*c*) with flowing cells colour-coded by time (electronic supplementary material, movie S1). (*d*) Projection of 1500 time-frames (17 ms interval) acquired in live 3–4 wpf *casper* fish injected intracardially with 10 KDa dextran conjugated with Alexa-647 (A647) (*n* = 8). Vertebrae, arteries and veins were identified as in (*c*) (electronic supplementary material, movie S3). (*e*) Rostral-caudal distribution of arteries and veins in abdominal vertebrae, with position normalized to vertebra length, in the two blood flow models: *Tg(-6.0itga2b:EGFP)* and dextran-A647. (*f*) Combination of all vertebrae and all samples shown in (*f*) in a single plot. (*g*) Schematic of the organization of arteries and veins in the developing SC vasculature. (CC, central canal). (*h*) Frequency of vertebrae that present each combination of number of arteries and veins. (*i*) Distance in µm of each artery/vein from the closest rostral artery/vein (each dot represents a vessel).

In the more developed vasculature, we also observed larger vessels connecting the internal vessels and the external network (arrowheads in figure 1*i*). To determine the arterial/venous identity of the connecting vessels and its distribution in relation to the vertebral column (figure 2*a*), we used live imaging to map the blood flow pattern in the juvenile SC (figure 2*b*). We used two models to image blood flow: (i) double transgenic line with labelled thrombocytes and ECs (*Tg(-6.0itga2b:EGFP);Tg(kdrl:HRAS-mCherry)*) (figure 2*c*–*c*″; electronic supplementary material, figure S1A,B; movie S1); and (ii) intracardiac injection of 10 KDa dextran conjugated to Alexa-647 in *casper* fish (figure 2*d*; electronic supplementary material, figure S2A,B, movie S2). With both approaches, we detected arteries on the ventral side and veins on the dorsal side in the v6-v12 abdominal region analysed (figure 2*e*), except for a few veins located ventrally (figure 1*c*; electronic supplementary material, movies S3, S4). Arteries were preferentially located at the centre of vertebrae, whereas veins were more widely distributed (figure 2*f*). Arteries and veins alternated along the rostral–caudal axis (figure 2*c,g*), but this pattern was not fixed, as adjacent arteries or adjacent veins were detected in several samples (electronic supplementary material, figure S1D, S2C). Most vertebral segments presented 1 artery and 1 vein (30%), but a high frequency of vertebrae had no arteries (21–26%) or no veins (18–28%) or neither (12–16%) (figure 2*g*). However, we may have missed some vessels due to limitations in our imaging conditions. The distance between arteries or veins also showed a high variability (figure 2*i*), but was on average higher than the average size of vertebrae (electronic supplementary material, figures S1E, S2D). This analysis reveals a dorsal–ventral segregation of veins and arteries in the zebrafish SC, with low correlation with the vertebral segmentation.

### 2.2. Spinal cord vasculature in adult zebrafish

**Figure 3.**
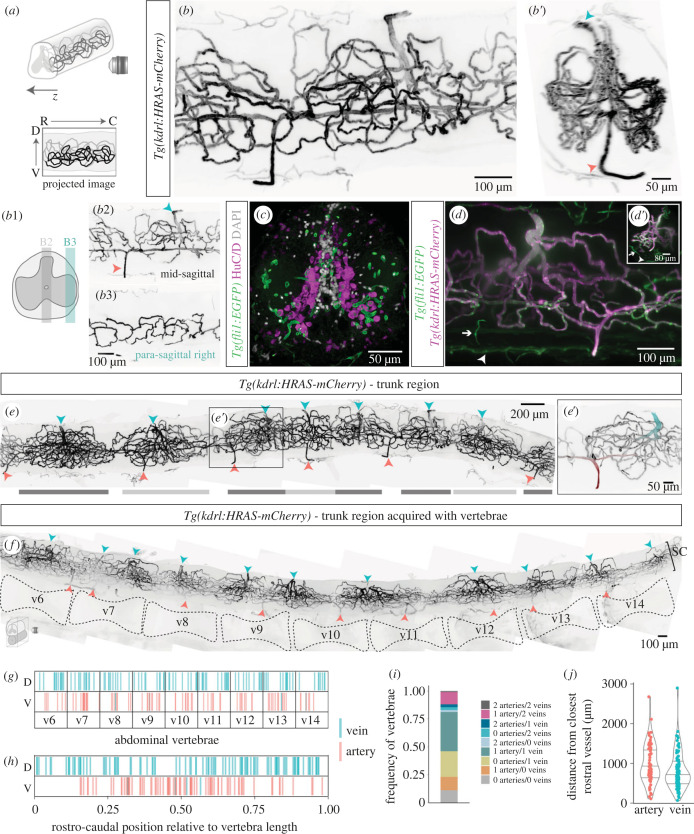
Organization of the spinal cord vasculature in adult zebrafish. (*a*) Schematic of light sheet image acquisition of wholemount cleared SCs (R, rostral; C, caudal; V, ventral; D, dorsal). (*b*) Wholemount SC with labelled ECs (*Tg(kdrl:HRAS-mCherry)*), with transversal view in *b*′. Arrowheads label arteries in red and veins in blue. *b*1. Schematic of the 100 µm regions projected in *b*2,*b*3. (*c*) Transverse SC section (*Tg(fli1:EGFP)^)^* co-labelled for HuC/D^+^ neurons and nuclear DAPI staining. (*d*) Whole SC (*Tg(fli1:EGFP;Tg(kdrl:HRAS-mCherry)*) with external *fli1^+^/kdrl^−^* vessels (arrows). (*d*′) Inset with transversal view of the image. (*e*) Composite of the trunk SC vasculature (*Tg(kdrl:HRAS-mCherry)*. Clusters of vessels are identified by alternating dark and light grey bars. (*e′*) Magnification of the region in (*e*), highlighting an artery in red with two branches and smaller calibre than the vein labelled in blue. A capillary cluster converges to the vein. (*f*) Composite of the trunk SC vasculature (*Tg(kdrl:HRAS-mCherry)* acquired with vertebrae. (*g*) Rostral–caudal distribution of arteries and veins in abdominal vertebrae, with position normalized to vertebra length. (*h*) Combination of all vertebrae and all samples shown in (*h*) in a single plot. (*i*) Frequency of vertebrae that present each combination of number of arteries and veins. (*j*) Distance in µm of each artery/vein from the closest rostral artery/vein (each dot represents a vessel).

We next characterized the adult SC vasculature in cleared wholemount samples with labelled ECs (*Tg(kdrl:HRAS-mCherry)*) (figure 3*a,b*). This analysis revealed a complex vascular network with preferential grey matter distribution and ventral and dorsal vessels connecting the internal vasculature with the external plexus (figure 3*b*′). Based on the blood flow data from juvenile fish and their morphology (arteries have smaller lumens compared to veins [26]), the ventral vessels were arteries that entered towards the centre of the tissue and ran along the central canal and branched into the central and lateral tissue (figure 3*b*1–3). These branches created a capillary network near neurons (figure 3*c*) and were collected by large dorsal veins. We were unable to detect vessels entering laterally, even with a broader endothelial marker (*Tg(fli1:EGFP* [27,28]) (figure 3*d*,*d*′), suggesting that the zebrafish SC was only supplied by a central system. These findings indicate that the peripheral system (vasocorona) described in the human SC [29] is absent in zebrafish.

A wider perspective of the SC revealed a segmented vasculature (figure 3*e*), with clusters of vessels that coincided with the position of veins, identified by the larger calibre when compared with arteries (figure 3*e*′). To determine if the distribution of arteries, veins and venous-associated clusters were correlated with the position of vertebrae we acquired SC images with the vertebrae still attached (figure 3*f*; electronic supplementary material, figure S3A–C). The quantification of the position of arteries and veins relative to vertebrae showed a similar pattern to that observed in juveniles: higher frequency of arteries at the centre of vertebrae and more dispersed distribution of veins (figure 3*g,h*); dorsal–ventral separation of veins and arteries (figure 3*g,h*), with some cases of ventral vessels with venous morphology (electronic supplementary material, figure S3D); higher frequency of vertebrae with 1 artery and 1 vein (35%), but many vertebral segments lacked arteries (26%) or veins (14%) or both (11%) (figure 3*i*; electronic supplementary material, figure S3E); high variability in the distance between vessels (figure 3*j*), on average higher than the mean vertebrae size (electronic supplementary material, figure S3F). These results suggest that the arterial–venous distribution established in juvenile stages is maintained in the adult SC and the vasculature grows in complexity in regions where veins are present.

To determine if the SC vascular density increased with body size in adult fish, we measured the vascular area in a SC section and compared with the fish body length (electronic supplementary material, figure S3G), which revealed a positive correlation (electronic supplementary material, figure S3H). To investigate if the sex of the fish played an influence in body length, SC size and/or vascular density, we analysed these parameters in females and males separately (electronic supplementary material, figure S3I–K). These data revealed similar body length, SC dorso-ventral size and vascular area between females and males at the age analysed (10–12 mpf), suggesting that sex has a limited effect on body size and SC vascularization at this age.

**Figure 4.**
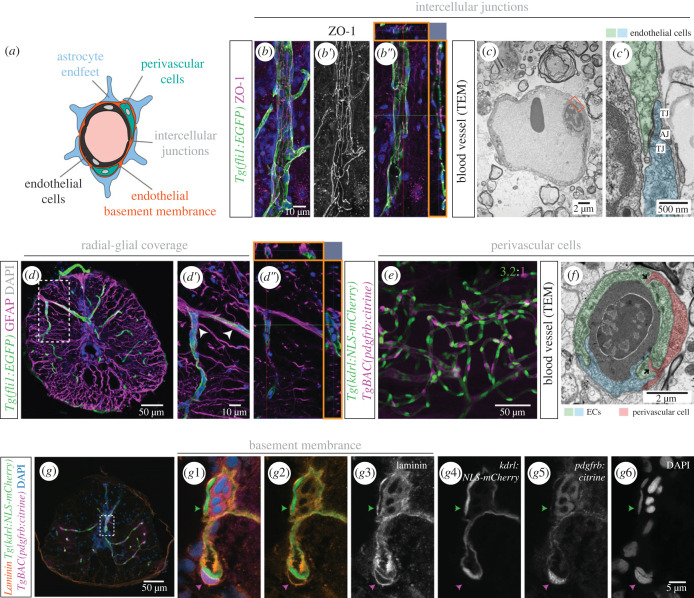
Organization of the blood-spinal cord barrier in adult zebrafish. (*a*) Schematic of BSCB components. (*b*-*b*″). *Tg(fli1:EGFP)* SC section, co-stained with the tight junction marker ZO-1 (*b*′). Orthogonal views (orange squares in *b*″) shows close proximity between ZO-1 and *fli1:EGFP*. (*c*-*c*′). TEM image of a blood vessel, with a magnification of the orange box in (*c*′) showing intercellular junctions between ECs: tight junctions (TJ) and adherens junctions (AJ). (*d–d*″). Transverse SC section labelled ECs *Tg(fli1:EGFP)* and radial glial cells (anti-GFAP antibody) with projections adjacent to ECs (arrowheads in (*d*′) and orthogonal views in orange squares in (*d*″)). (*e*) Wholemount SC with labelled endothelial nuclei (*Tg(kdrl:NLS-mCherry)*) and pericytes (*TgBAC(pdgfrb:citrine)*) (3.2 ECs per perivascular cell ± 0.59, *n* = 7). (*f*) TEM image of a cross-section of a blood vessel, showing two contacting ECs (in blue and green) and an associated pericyte (in red). (*g*) Transverse SC section with labelled endothelial nuclei (*Tg(kdrl:NLS-mCherry)*), pericytes (*TgBAC(pdgfrb:citrine)*), co-labelled with an anti-laminin antibody to identify the basement membrane (BM) and DAPI-labelled nuclei. (*g*1–*g*6) Magnifications of the region labelled in (*g*) showing the separate channels. The green arrowhead points to the BM over an endothelial nucleus and the magenta arrowhead identifies a pericyte embedded in the endothelial BM.

We also investigated the presence of BSCB components: ECs are sealed by intercellular junctions and have a selective transport system; the endothelial layer is covered by pericytes, enveloped in a basement membrane (BM) and wrapped by astrocyte endfeet [30] (figure 4*a*). SC blood vessels displayed tight junctions (identified using tight junction marker ZO-1) (figure 4*b*–*b*″), and also adherens junctions, observed with transmission electron microscopy (TEM) (figure 4*c*,*c*′). We used an anti-GFAP antibody to label glial cells and detected GFAP^+^ projections adjacent to ECs, indicating that glial cells encircle blood vessels (figure 4*d*–*d*″). Using the transgenic line *Tg(pdgfrb:citrine*), we detected solitary *pdgfrb*^+^ cells in close association to ECs (figure 4*e*), with a similar distribution to a previous study [25]. We identified these perivascular cells as pericytes based on their isolated distribution and association with small diameter vessels [31]. The zebrafish SC exhibited a high ratio of *pdgfrb*^+^ cells to ECs (1 : 3.2; data from sham condition in figure 8*i*), comparable to the ratio described in the mammalian central nervous system (CNS) [32]. A TEM image also revealed peg–socket junctions between a perivascular cell and ECs (figure 4*f*). Finally, we examined the presence of a BM using an anti-laminin antibody, which showed the BM enveloping not only ECs, but also *pdgfrb*^+^ cells (figure 4*g*–g6), further supporting the identification of these cells as pericytes [33]. This characterization reveals that, as in mammals, the adult zebrafish SC displays blood vessels with specialized modifications associated with a mature BSCB.

### 2.3. Blood vessels rapidly re-vascularize the injured spinal cord

**Figure 5.**
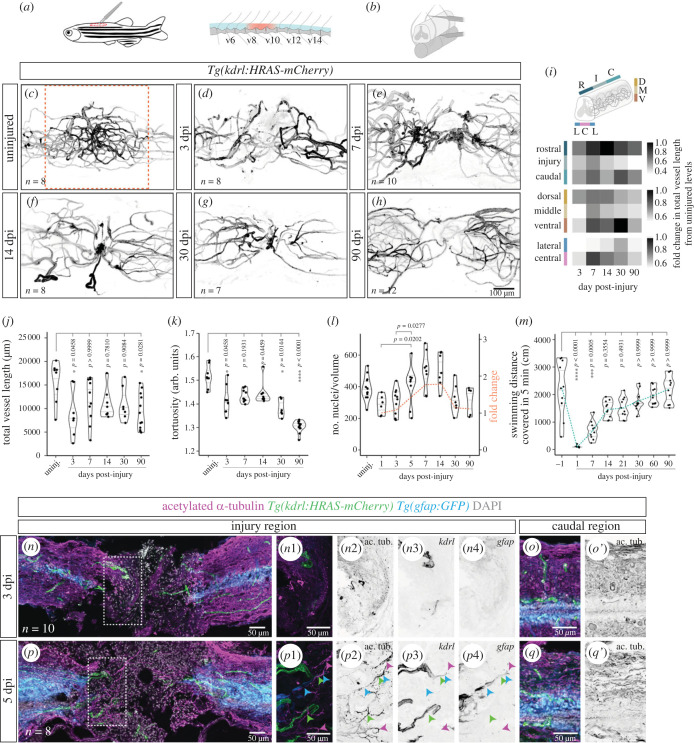
Changes in vascular distribution and morphology in response to spinal cord injury. (*a*) Schematic of the location of the SC injury in adult zebrafish. (*b*) Schematic of a contusion injury, using forceps to compress the SC between neural arches. (*c–h*) Wholemount SCs with labelled endothelial cells (*Tg(kdrl:HRAS-mCherry)*) in uninjured and 3, 7, 14, 30 and 90 dpi. Images are of different animals representative of each time-point. (*i*) Fold change in total vessel length relative to uninjured levels over time post-injury in different regions along the rostral-caudal axis, the dorsal–ventral axis and the left-right axis. (*j*) Average vessel length over time post-injury. (*k*) Average vessel tortuosity (vessel length/euclidean distance) over time post-injury. (*l*) Number of endothelial nuclei over time post-injury. The orange dashed line indicates the fold change in the number of nuclei relative to 1 dpi (right-side axis). (*m*) Swimming distance covered in an open field test from 1 day before injury to 90 dpi (green dashed line represents the mean value). (*n,p*) Longitudinal sections of transected SCs with labelled axons (anti-acetylated alpha-tubulin antibody), ECs (*Tg(kdrl:HRAS-mCherry)*), glial cells (*Tg(gfap:GFP)*) and nuclear DAPI staining, at 3 (*n*) and 5 dpi (*p*). *n*1–*n*4. Magnifications of box in (*n*) showing blood vessels, but not axons or glial cells, present in the injured region at 3 dpi. *p*1–*p*4. Magnifications of box in (*p*) showing axons adjacent to blood vessels (green arrowheads), glial projections (blue arrowheads) or alone (magenta arrowheads). (*o*,*o*′), (*q*,*q*′). SC region 2 mm caudally to the injury. Statistical tests: Kruskal–Wallis test followed by Dunn's multiple comparisons *post hoc* test relative to uninjured control (*j,k,m*) or between all conditions (*l*) (*p*-values > 0.05 in (*l*) are not shown).

To examine how blood vessels respond to a SCI, we performed a contusion injury in adult zebrafish (figure 5*a,b*) and imaged the vasculature in wholemount samples (*Tg(kdrl:HRAS-mCherry)*) at different days post injury (dpi). A contusion injury was used to mimic the effects of a traumatic SCI in patients, namely the widespread damage of the tissue and the disruption of axonal tracts and blood vessels [1]. To quantify vessel length and morphology, we used a three-dimensional image analysis workflow [34] (electronic supplementary material, figure S4). The continuous vasculature in the uninjured SC (figure 5*c*) was interrupted by the injury and vessels remained severed at 3 dpi (figure 5*d*). New vessels rapidly re-vascularized the lesioned region and formed agglomerates by 7 dpi (figure 5*e*). Some of the vessels in the agglomerates were larger and tortuous, features of dysfunctional vessels. In addition, the vessels from the rostral and caudal sides appeared to have anastomosed at the injury centre, suggesting that circulation was reconnected. The vessel knots were still visible at 14 dpi, but few were detected at 30 and 90 dpi (figure 5*f–h*). Vessel recovery was not homogeneous in all domains (figure 5; electronic supplementary material, figure S4J–Q). Along the rostral–caudal axis, the injury site remained impaired at 90 dpi (figure 5*i*, top panel) and along the dorsal–ventral axis the recovery was lowest in the middle region, where vessel density was highest pre-injury (figure 5*i*, middle panel). Along the left-right axis, the central region (with highest vessel density pre-injury) showed a better recovery after injury than the lateral regions, suggesting that new vessels grow preferentially near the central canal area (figure 5*i*, bottom panel). However, vessel density remained lower than the control in both central and lateral regions at 90 dpi (electronic supplementary material, figure S4M,Q). Overall, total vessel length failed to recover to pre-injury levels (figure 5*j*) and the complexity of the vasculature was also reduced post-injury, as reflected by the presence of more linear vessels at 30 and 90 dpi (figure 5*k*). We also examined the distribution of arteries and veins at 90 dpi, to determine if the injury preferentially occurred in regions of high vessel density associated with veins (electronic supplementary material, figure S5). However, we observed a high variability in the distance of arteries and veins from the injury, suggesting that the injury may affect regions with different vessel density depending on the sample.

We also evaluated the vascular recovery in terms of EC number (*Tg(kdrl:NLS-mCherry)*). The quantification of endothelial nuclei showed similar dynamics to the quantification of vascular length: the number of ECs decreased after the injury, was followed by a recovery at 7 dpi with the formation of vessel agglomerates and decreased again from 30 dpi onwards (figure 5*l*; electronic supplementary material, figure S6). The reduction in vascular complexity and cell number observed in later time-points suggests that during the rapid repair process vessels are formed in excess and some are dysfunctional and later are removed or remodelled.

The early phase of vessel regrowth also occurred in parallel with the recovery of the swimming capacity (figure 5*m*). To examine how re-vascularization was coordinated with axons (acetylated alpha-tubulin) and glial bridge cells (*Tg(gfap:GFP)*) [35,36], we used a SC transection model [17], in which axons and glia are eliminated in the injury site (electronic supplementary material, figure S7A,B), resulting in complete paralysis followed by gradual motor recovery over six weeks [36]. Both contusion and transection models showed similar vascular dynamics in response to injury (electronic supplementary material, figure S7C–H). After transection vessels were detected close to the injury site at 3 dpi, when axons and glial cells were still absent (figure 5*n*–*o*′). At 5 dpi, when spared or growing axons were observed in the injured tissue, some axons were detected near blood vessels, with or without accompanying glial projections (figure 5*p–q*′). The aligned distribution between vessels and axons was not observed in later time-points (electronic supplementary material, figure S8). These observations point to a possible early association between vessels and axons.

In summary, an initial phase of rapid endothelial growth with formation of tortuous vessels that agglomerate in the central region, where the presence of progenitor cells and/or surrounding neurons possibly influences the vascular response. This vasculature appears to be optimized later, resulting in a simplified version of the vascular network, with fewer and more linear vessels than in control SCs.

### 2.4. Re-establishment of barrier properties in the repaired vasculature

**Figure 6.**
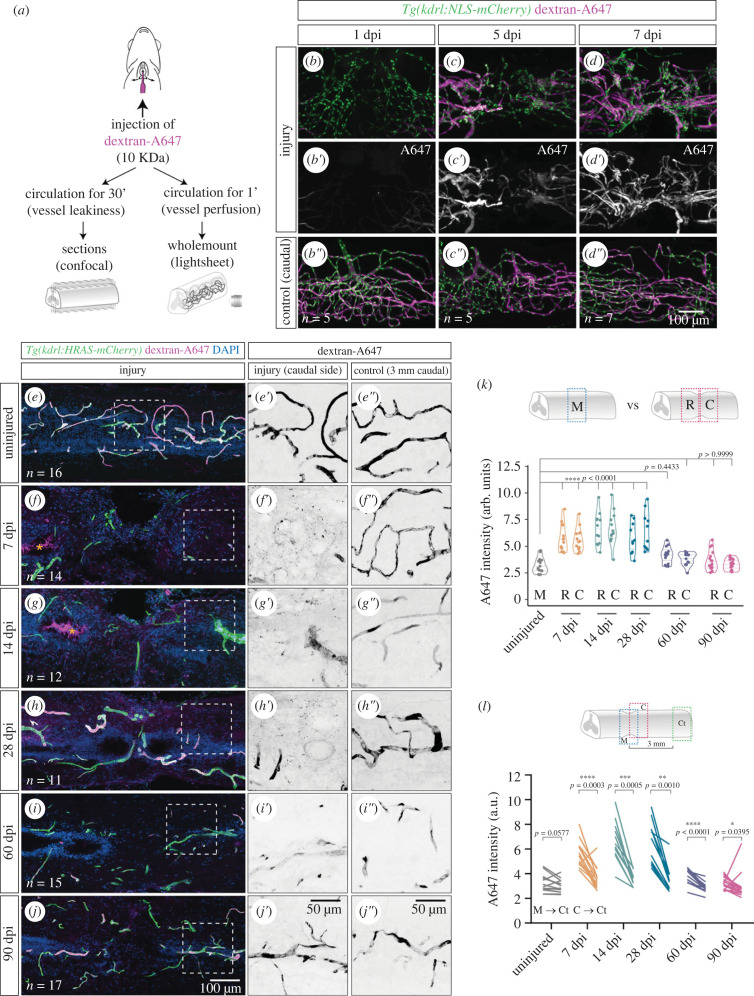
Re-establishment of the blood-spinal cord barrier after injury. (*a*) Schematic of the vessel perfusion/permeability assay with 10KDa dextran-Alexa647 (A647), adapted from [37]. (*b–d*″). Wholemount SCs with labelled endothelial nuclei (*Tg(kdrl:NLS-mCherry)*), perfused with dextran-A647 for 1 min and analysed at 1, 5 and 7 dpi. (*b*′–*d*′). A region 2 mm caudally to the injury is used as control of the injection (*b*″–*d*″). (*e–j*) Longitudinal sections of contusion-injured SCs with labelled ECs (*Tg(kdrl:HRAS-mCherry)*), perfused with dextran-A647 for 30 min and with DAPI-stained nuclei. Orange asterisks highlight the accumulation of dextran-A647 in the central canal in *f*,g. Magnified views of the dextran-A647 signal in the caudal side of the injury are shown in *e*′–*j*′. and a control region 3 mm caudal to the injury is shown in *e*″–*j*″. (*k*) Quantification of extravascular intensity of dextran-A647 in the rostral (R) and caudal (C) side of the injury and compared to the middle region (M) of uninjured SCs. (*l*) Comparison of dextran-A647 extravascular intensity in the caudal side of the injury (or a corresponding region in uninjured SCs) with a control region 3 mm caudal to the injury. Each line shows the change between the two regions in individual SCs. Statistical tests: Kruskal–Wallis test followed by Dunn's multiple comparisons *post hoc* test relative to uninjured control (*k*) and Wilcoxon matched-pairs signed rank test (*l*).

To determine if the new vessels were functional, i.e. if vessels were perfused and developed a BSCB, we injected the 10 KDa dextran Alexa Fluor 647 (A647) into circulation (figure 6*a*) [37]. This tracer dye is size-restricted from the CNS [38]. To assess perfusion of new vessels, we allowed the dye to circulate for 1 min before collecting and imaging the wholemount SC (figure 6*b–d″*). We confirmed that the injection was successful by acquiring a region caudal to the injury (figure 6*b*″–*d*″). At 1 dpi the tracer was absent from most vessels in the injured region (figure 6*b*,*b*′), suggesting that vessels were not yet perfused or the dye was not retained inside the vessels. At 5 dpi, the dye was observed in vessels closer to the injury (figure 6*c*,*c*′) and by 7 dpi most vessels were filled with tracer (figure 6*d*,*d*′), indicating that the growing vessels had successfully fused and blood was flowing across the injury site.

To determine if the BSCB was re-established in the repaired vasculature, we allowed the dextran-A647 to circulate during 30 min before collecting the SC. The dye is expected to diffuse into the tissue over time if the vessels are leaky [39]. We analysed the distribution of dextran-A647 in sections in uninjured SCs and from 7 dpi (when new vessels are perfused) until 90 dpi (figure 6*e–j*). The intensity levels of dextran-A647 were measured in the tissue parenchyma in the rostral and caudal injury sides or in control regions (uninjured SC or region distant from the injury) (electronic supplementary material, figure S9A-B′). Extravasated dye was not detected in uninjured SCs (figure 6*e*,*e*′) or in control regions 3 mm caudal to the injury (figure 6*e*″–*j*″), indicating that the barrier properties of vessels were intact. By contrast, speckles of dye were detected in the extravascular tissue at 7, 14 and 28 dpi (figure 6*f*,*g*,*f*′–*h*′), revealing that vessels are still permeable at these time-points. This speckled distribution was only present in the injured tissue when dextran-A647 was injected (electronic supplementary material, figure S9C-D′), indicating that the signal is specific for the extravasated dye. The quantification of A647 intensity in the parenchyma confirmed that the dye levels were significantly increased in the injured region until 28 dpi when compared with control conditions (figure 6*k,l*). Moreover, this quantification revealed that A647 leakage is comparable between the rostral and the caudal injury sides. From 60 dpi, the parenchymal A647 levels had returned to uninjured levels (figure 6*i,j″*,*k*), although still above caudal levels (figure 6*l*). This indicates that the barrier properties of the repaired vasculature are re-established between 28 and 60 dpi. These results reveal that the BSCB development is a delayed process when compared with new vessel formation, suggesting that the SC tissue remains exposed to circulating compounds and cells for a prolonged period.

### 2.5. Angiogenesis contributes to new vessel formation

**Figure 7.**
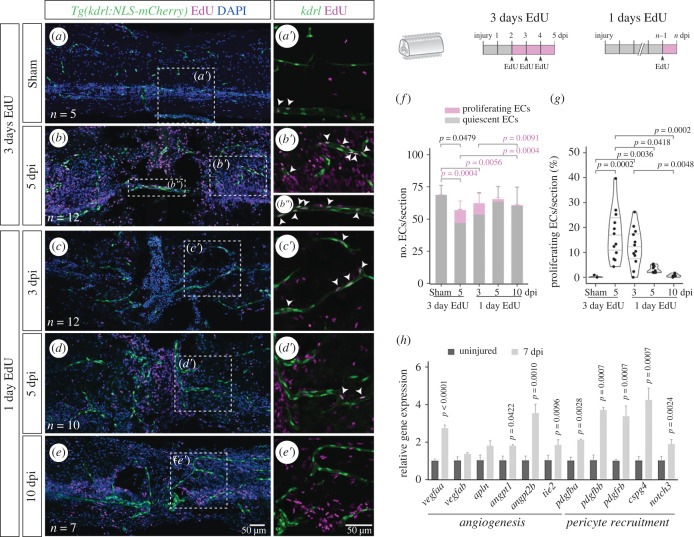
Endothelial proliferation during vascular repair. (*a–e*) Longitudinal SC sections with labelled ECs (*Tg(kdrl:NLS-mCherry)*), EdU staining and nuclear DAPI staining, with magnified views (*a*′–*e*′) showing proliferating ECs (arrowheads). Repeated EdU injections were performed three consecutive days before SC collection at 5 days after sham/spinal cord contusion injury (*a,b*). Single EdU injections were administered 1 day before SC collection at 3, 5 and 10 dpi (*c,d,e*). (*f*) Total number of ECs per section, grouped as proliferating (EdU^+^) or quiescent (EdU^−^) cells. (*g*) Fraction of proliferating ECs (EdU^+^ ECs / total ECs) per section. (*h*) Relative gene expression in uninjured (*n* = 6) and 7 dpi (*n* = 2) SCs, measured by qPCR, standardized to *gapdh* and normalized to the uninjured mean. Data represent mean ± s.d. Statistical tests: Kruskal–Wallis test followed by Dunn's multiple comparisons *post hoc* test (*f*,*g*); two-tailed unpaired *t*-test (O); (*p*-values > 0.05 are not shown).

To investigate how the vasculature is repaired, we focused on the phase of new vessel formation. From 1 to 7 dpi, the number of ECs showed an almost twofold increase (from 284.5 ± 60.4 to 502.6 ± 120.3) (figure 5*l*). This fold change suggests that endothelial proliferation and/or migration contribute to vessels regrowth into the injured tissue. To assess the contribution of endothelial proliferation to the formation of new blood vessels, we performed EdU incorporation after SC contusion/sham injury. Following EdU injection in three consecutive days (2–4 dpi) we observed a significant increase in the number of proliferating ECs in 5 dpi SCs, when compared with sham injuries (figure 7*a*,*b*″*f*). The proportion of proliferating ECs increased from 0.2% ± 1.0 in sham animals to 15% ± 7.7 at 5 dpi (figure 7*g*), suggesting that endothelial proliferation contributes to the repair of damaged blood vessels.

To examine the dynamics of EC proliferation, we performed single-pulse EdU incorporation one day before SC collection at 3, 5 and 10 dpi (figure 7*c–e′*). A peak of proliferating ECs was observed at 3 dpi and gradually decreased until 10 dpi (figure 7*f,g*). These data reveal that an early and transient proliferative response contributes to the repair of the injured vasculature.

To gain insight into the signalling pathways involved in the formation of new vessels, we analysed the expression of genes involved in vessel growth and maturation in injured SCs (figure 7*h*). Genes associated with angiogenesis (*vegfaa*, *angpt1*, *angpt2b*, *tie2*) and pericyte recruitment (*pdgfba*, *pdgfbb,* *pdgfrb,* *cspg4*, *notch3*) were upregulated at 7 dpi. These changes point to possible pathways involved in vascular re-growth, while the activation of the Pdgf pathway may be involved in the recruitment of pericytes to the repaired vasculature.

### 2.6. Pericyte recruitment during vessel stabilization

**Figure 8.**
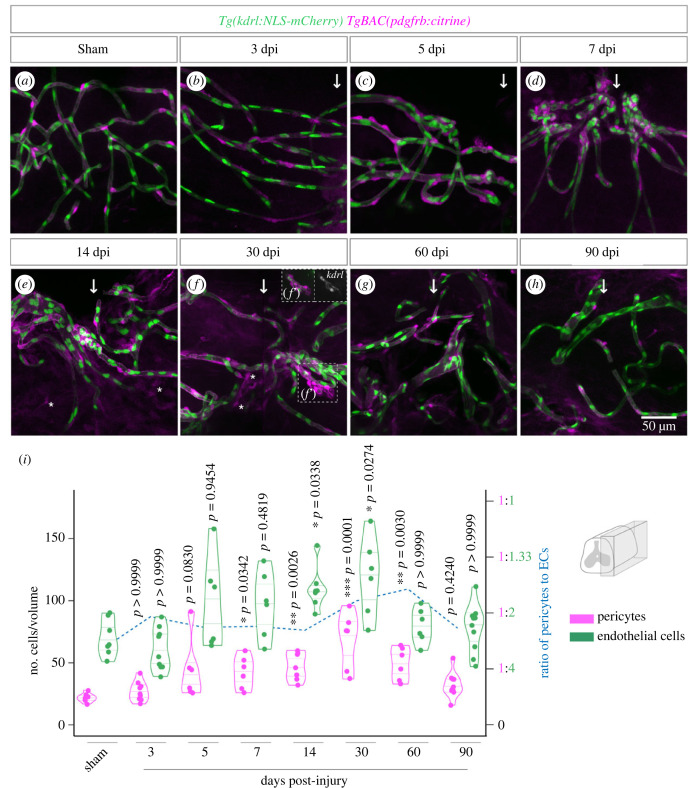
Pericyte recruitment during vascular repair. (*a–h*) Lateral region (90 µm depth) of wholemount SCs with labelled endothelial nuclei (*Tg(kdrl:NLS-mCherry)*) and pericytes (*TgBAC(pdgfrb:citrine)*) after sham injury and between 3 and 90 days post-contusion injury. The arrows identify the position of the injury. Asterisks identify cells with low *pdgfrb:citrine* expression not associated with vessels. Region in (*f*′) shows a vessel with low levels of *kdrl:NLS-mCherry* and associated pericytes (in inset, *kdrl* levels are enhanced relative to the main figure). (*i*) Total number of pericytes and ECs over time post-injury. Ratio of pericytes to ECs is shown as a dashed blue line (right-side axis). Statistical tests: Kruskal–Wallis test followed by Dunn's multiple comparisons *post hoc* test relative to uninjured control (*i*).

To determine if the formation of new vessels was coordinated with the recruitment of support cells, including pericytes, we analysed transgenic zebrafish with labelled pericytes and endothelial nuclei (*Tg(pdgfrb:citrine)*; *Tg(kdrl:NLS-mCherry)*) up to 90 days after a SC contusion/sham injury. We observed a higher density of pericytes surrounding repairing vessels, with a gradual increase from 3 dpi until 30 dpi, with significantly higher numbers from 7 to 30 dpi when compared with a sham injury (figure 8*a–h*,*i*). We detected proliferating pericytes in early time-points, which may contribute to the increase in pericyte number (electronic supplementary material, figure S10). From 30 dpi, the number of pericytes decreased until the last time-point analysed, 90 dpi, when pericytes reached pre-injury levels. A similar dynamic was observed for the number of ECs, but pericytes showed a proportionally higher increase, which was reflected in the ratio of pericytes to ECs (blue dashed line in figure 8*i*). The highest pericyte density was observed at 30 and 60 dpi, which corresponded to the period when the BSCB was being re-established. By 90 dpi, when the barrier had recovered, pericyte density returned to homeostatic levels.

These data show that repaired vessels have increased pericyte density for an extended period. After the remodelling and maturation of the new vasculature, the coating of vessels by pericytes returns to the homeostatic density, possibly contributing to the re-establishment of the barrier.

The majority of cells labelled by the *pdgfrb:citrine* transgene were associated with vessels and were identified as pericytes based on the morphology and density. A population of *pdgfrb*^+^ cells detached from vessels has also been described at the injury core in mammalian [10] and larval zebrafish SCs [25]. Although we detected a few non-perivascular cells with low *pdgfrb:citrine* expression in the injured region (figure 8*e,f*; electronic supplementary material, figure S11), we cannot confirm if this population is indeed present in the adult zebrafish SC due to the low signal levels of this reporter line.

To determine if other BSCB components were also present in repaired blood vessels, we analysed radial glial processes (*Tg(gfap:GFP)*) and tight junctions (ZO-1) associated with vessels at the injury site at 28 dpi (when vessels are still leaky) and 60 dpi (when the barrier is re-established). The fraction of vessels with no/low *gfap:GFP* coverage was comparable between 28 and 60 dpi (electronic supplementary material, figure S12), suggesting that the recovery of the barrier is not strongly correlated with the level of associated radial glial processes. Likewise, most vessels at the injury site at 28 dpi had associated ZO-1 staining, at a similar level to vessels at 60 dpi (electronic supplementary material, figure S13), suggesting that tight junctions are formed in repaired vessels even before the barrier function is fully recovered.

**Figure 9.**
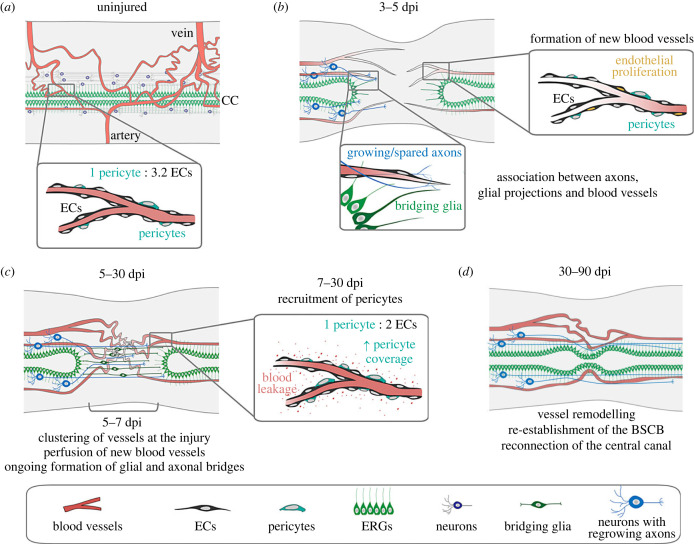
Model of vascular repair in the zebrafish spinal cord. (*a*) Organization of the SC vasculature in adult zebrafish. Ventral arteries give rise to vessels that run along the ependymal canal and branch as grey matter capillaries. These capillaries aggregate around collecting dorsal veins, forming vascular clusters. Small calibre vessels are supported by pericytes. (*b*) SCI triggers early endothelial proliferation. New blood vessels rapidly invade the injured tissue and are observed in proximity of glial projections and growing/spared axons. (*c*) Rostral and caudal growing vessels anastomose in the injured region and become perfused between 5 and 7 dpi, as the glial and axonal bridges are formed. The initial vascular repair creates a network of irregular vessels. Pericytes are recruited to new blood vessels from 7 dpi until 30 dpi, but vessels remain permeable during this period. (*d*) Between 30 and 90 dpi the vascular network is remodelled and the BSCB is re-established, allowing for the long-term and functional vascularization of the regenerated SC.

Taken together, these results reveal that the regeneration of the zebrafish SC also includes the repair of the vascular system, with an early angiogenic phase, followed by intermediate/late phases of pericyte recruitment and vessel stabilization and remodelling (figure 9).

## 3. Discussion

Here, we report the first detailed characterization of the SC vasculature in developing and adult zebrafish and show that blood vessels are successfully repaired after SC injury in this regenerative organism.

The zebrafish SC is vascularized in a late larval stage [20,22]. Here, we propose that the size of a fish, and in particular the size of the SC, determines the timing of vascular ingression, more than the fish age. Even though the ages analysed in the different studies are different, our hypothesis is supported by the fact that the average SC area at the start of vessel entry is comparable between our study (8378 ± 2106 µm^2^ in 35–42 dpf fish) and the Matsuoka *et al*. study (8268 ± 1673 µm^2^ in 18–20 dpf fish; *n* = 3, measured in transverse sections from figures and movies in [20]). The age difference between studies could be explained by differences in husbandry conditions, such as type and availability of food and tank density, which can impact growth speed [40].

The SC area at which vessels start invading the spinal tissue corresponds to a 50 µm radius, which is consistent with the distance of most cells to its nearest capillaries (less than 100 µm) [41,42]. Below this distance oxygen diffusion from the PNVP could be sufficient to reach the centre of the tissue and above this distance cells could start activating hypoxic signals and induce angiogenesis. Further work is needed to determine if the tissue becomes hypoxic and which cells and pathways are involved in attracting vessels into the SC. Neural-derived VEGF-A is involved in the vascularization of the developing avian neural tube [43]. Spinal neuron- and radial glia-derived sFlt1 and Vegfab were also shown to affect the PNVP in earlier stages in zebrafish [20,22,44] and these cell types and pathways may also be involved in promoting vessel entry in later stages.

Our findings also reveal that vessels entering and spreading into the SC are accompanied by pericytes, possibly derived from the PNVP perivascular population (this study and [25]). Pdgfrb^+^ pericytes are possibly attracted to nascent vessels by Pdgfb-expressing ECs, as described during mouse brain development [45]. Brain pericytes also proliferate and help regulate EC number and BSCB assembly as the vasculature grows [31,45]. We also observed pericyte expansion in proportion to the increase in endothelial number, with a ratio of 1 pericyte to 3 ECs that is maintained until adulthood. Whether pericytes contribute to the establishment of the BSCB in zebrafish remains to be uncovered.

We also describe the ventral-to-dorsal circulatory route in the juvenile SC. However, it is still unclear how the arterial-venous pattern is established and if signals such as Cxcl12-Cxcr4, BMPs, Ephrin-Eph or blood flow are involved [46].

Our analysis of the adult SC vasculature showed a similar pattern of dorsal and ventral vessels entering the SC. Although we were unable to confirm the arterial-venous identity with molecular markers, we defined the ventral vessels as arteries and the dorsal vessels as veins based on the blood flow characterization in juveniles, except for some ventral vessels with venous morphology. Arteries enter ventrally, branch extensively to create the complex capillary network that irrigates the grey matter and converge again to large veins located dorsally. The arterial-venous organization in the zebrafish SC differs from the human SC, where arteries and veins are found both dorsally and ventrally [29,47]. The zebrafish SC also lacks the peripheral irrigation from a vasocorona described in the human SC [29], suggesting that a peripheral source is not necessary to irrigate the small-sized SC.

The adult zebrafish SC has a developed BSCB, with associated perivascular cells and radial glial cells, tight junctions and other intercellular junctions between ECs. The presence of these components is indicative of a controlled transit of cells and molecules between blood and nervous tissue. We identified the perivascular cells labelled by the *pdgfrb:citrine* transgene as pericytes, based on the isolated distribution, association with small vessels and BM contiguous with ECs. It is possible that other mural cells (i.e. vascular smooth muscle cells) or different pericyte subtypes [48] are present in the adult SC, but we have not assessed these populations.

In this study, we also provide a detailed temporal and spatial description of the vascular response to SCI. We show that the lesion severs blood vessels, but new vessels quickly reappear in the injury epicentre, from both rostral and caudal regions, and before glial projections and axonal sprouts. Endothelial proliferation contributes to the re-vascularization of the injured tissue, but is temporally restricted to early time-points, suggesting that endothelial migration may play a role afterwards. Other cell types, such as lymphatic ECs, may also transdifferentiate to give rise to blood ECs in response to injury [49], but additional lineage tracing studies are needed to assess this possibility.

The pathways involved in this angiogenic response are also unclear. We detected the injury-dependent expression of genes in pathways associated with angiogenesis (Vegf, Angiopoietins), but further work is needed to assess their role in vascular repair. This knowledge will also be important to create tools to deplete blood vessels and assess their role in SC regeneration, either to provide support for the tissue or as a source of pro-regenerative signals for stem cells [50,51] or axons [52]. We detected blood vessels in the injury gap before glial cells and axons and some vessels were later associated to spared or growing axons, raising the possibility that vessels act as pioneers for regrowing axons.

The invading vessels often travel together and create agglomerates at the injury core that fuse the rostral and caudal sides of the vascular network and re-establish circulation. Some of the initial vessels have a tortuous morphology, which is associated with hyperpermeability [53] and can be a result of high local VEGF-A levels (which we detected in the injured SC) or the type of VEGF-A isoforms expressed [54]. Consistent with the presence of tortuous/dysfunctional vessels, vessels were permeable to a fluorescent dextran until 30 dpi. However, the tortuous vessels were transient, suggesting that the repaired vasculature is remodelled to remove the dysfunctional vessels and optimize circulation. By 90 dpi, when most atypical vessels had been removed, vessel permeability had also decreased to pre-injury levels, indicating that the repaired spinal tissue is supported by long-term vessels with a functional BSCB. Nevertheless, the injured SC failed to recover the vascular distribution to pre-injury levels, particularly in regions that were highly vascularized before the injury, such as the grey matter surrounding the central canal. The recovery of vessel density in these regions may take longer than the time window analysed in this study.

The mechanisms underlying the recovery of the BSCB are still unclear, but pericytes are likely involved. New blood vessels were rapidly covered by pericytes, formed through proliferation and possibly migration of existing pericytes. The Pdgfrb/Pdgfb pathway is probably involved in pericyte recruitment, as we saw receptor and ligand upregulation in response to injury. Although pericytes started to be recruited early on, pericyte density only reached peak levels between 30 and 60 dpi, which corresponded to the period when vessels became less permeable. The gradual accumulation of pericytes may be important to promote the recovery of the barrier. Alternatively, it is possible that, despite being present in high density, early pericytes are unable to provide sufficient surface coverage of vessels, which can be correlated with vessel permeability [24]. Additional studies need to be performed to understand the role of pericytes in BSCB recovery, for example through regulation of endothelial transcytosis and astrocyte end feet function [55]. In addition, the analysis of glial coverage and tight junctions in repaired vessels revealed that these structures were present even before the re-establishment of barrier function. Further work is needed to modulate these structures, in order to analyse their role in the repair of the barrier and test how interfering with the barrier affects tissue regeneration and functional recovery.

In conclusion, this study establishes the vascular system as a new component of the regenerative program in the zebrafish SC, but further work is needed to understand how vascular repair is regulated and how blood vessels influence other components of the injury response. This work also highlights the benefits of performing SCI studies in adult zebrafish, since larval models miss the contribution of the vascular component.

## 4. Material and methods

### 4.1. Fish husbandry and zebrafish lines

Zebrafish (*Danio rerio*) lines were raised and maintained at 28.5°C in a 10/14 h dark-light cycle, according to FELASA recommendations [56]. Fish were fed a diet of once daily dry food (Zebrafeed, Sparos) and once daily live food (Artemia). Fish used in live imaging experiments were fed once daily with rotifers, which accelerated their growth relative to the dry food/Artemia diet. We used AB wild-type strain, *mitfa^w2/w2^; mpv17^a9/a9^* mutants (hereby designated *casper*) [57] and transgenic lines that were previously established: *Tg(kdrl:HRAS-mCherry)^s896^* [58]; *TgBAC(pdgfrb:citrine)^s1010^* [59];*Tg(kdrl:NLS-mCherry)^is4^* [60]; *Tg(fli1:EGFP)^y1^* [61]; *Tg(gfap:GFP)^mi2001^* [62]; *Tg(-6.0itga2b:eGFP)^la2^* [63].

### 4.2. Spinal cord injury and motor behaviour analysis in adult zebrafish

Adult zebrafish of either sex, between 4 and 12 month-old, were randomly assigned to experimental groups. Fish were anaesthetized by immersion in 0.6 mM tricaine (MS222, Sigma) in system water. Fish were placed with the left side up on a sponge to make a longitudinal incision on the side of the fish, midway between the base of the skull and the dorsal fin (v8–v10 abdominal region), and the muscle fibres were separated to expose the vertebral column. For the contusion injury, the SC was compressed dorsoventrally in the region between neural arches using forceps (Dumont #55, FST) [15]. For the transection injury, the SC was completely transected using fine scissors (no. 15000-03, FST) [17]. The sham injury was performed by making an incision at the side of the animal but leaving the SC intact. The wound was closed with forceps and the fish was placed in system tanks with 6–10 other injured fish in a ZebTEC Stand-Alone Toxicology Rack (Tecniplast). Only fish that exhibited complete paralysis the day after the injury (contusion or transection) were used for the experiments.

To evaluate the motor recovery after SCI, we performed an open field test [15]. Individual fish were placed in 9.5 × 16.5 cm tanks with system water with a light source underneath and allowed to acclimate for 5 min before recording the swimming behaviour for 5 min from a top view (Ikegami digital video camera). The swimming behaviour was tracked using the EthoVision XT 15.0 software (Noldus) to obtain the total distance travelled during 5 min. The same group of fishes were followed over the course of the trials. The obtained data were plotted using ggplot2 [64] and analysed statistically using GraphPad Prism 8.

### 4.3. General image acquisition and processing details

The systems, objectives and *z*-stack intervals are detailed in the subsections. The pinhole aperture was adjusted to keep the same optical slice thickness between the different channels. The laser intensities were kept at the same level between samples and conditions in the same experiment. Images were processed using Fiji [65] and Adobe Photoshop CS4 and figures were prepared in Adobe Illustrator CS4.

### 4.4. Tissue sectioning in juvenile and adult zebrafish

Fish were sacrificed in 15 mM tricaine and fixed in 4% paraformaldehyde (PFA) at 4°C overnight. In juvenile fish, the head was removed and the rest of the body was fixed; in adult fish, the vertebral column was dissected and placed in fixative. After fixation the sample was washed 3 × 5 min in PBS and, for adult fish, the SC was isolated from the vertebral column using forceps. The samples were equilibrated in sucrose solution (15% sucrose; 0.12 M phosphate buffer (PB) pH 7.2) at 4°C overnight, embedded in gelatine (7.5% gelatine; 15% sucrose; 0.12 M PB pH 7.2) at 37°C, frozen in isopentane cooled to −40°C in dry ice and stored at −80°C. In juvenile samples, the trunk region was cryosectioned in 25 µm-thick transversal slices collected in four alternating slide sets. Adult SCs were cryosectioned in 14 µm-thick transversal sections mounted in 6 alternating slide sets, or in 25 µm-thick longitudinal slices collected in a single slide.

### 4.5. Immunohistochemistry in sections and wholemount samples and image acquisition

To perform immunostaining in sections, the gelatine was removed from the cryosections using PBS heated to 37°C (4 × 5 min washes). After incubation with a blocking solution (1% BSA in PBS; 0.1% Triton X-100 in PBS (PBSTx)) for 1 hour at RT, the sections were incubated in primary antibody solution at 4°C overnight. After 5 × 5 min washes in PBSTx, the sections were incubated with the secondary antibody (1 : 1000 in blocking solution) and 1 µg ml^−1^ DAPI (#D9564, Sigma) for 2 h at room temperature (RT). Details of the primary and secondary antibodies used are described in electronic supplementary material, tables S1 and S2. After incubation with the secondary antibodies, the sections were washed in PBS and mounted in Mowiol solution (2.4 g Mowiol; 6 g glycerol; 6 ml dH2O; 12 ml 0.2 M Tris buffer pH 8.5) with #1.5 coverslips and allowed to dry overnight. Images of transversal sections shown in figures 1 and 2 were acquired in a Zeiss LSM 710 confocal microscope with 20X Plan-Apochromat dry objective. Images were acquired as *z*-stacks (four slices 1 µm apart). Images for the quantification of number of vessels (figure 1) were acquired in a Leica DM5000B wide-field fluorescence microscope mounted with a monochrome CCD camera, using a 20x HC PLAN APO objective. Acquisition started approximately at the heart section level and for at least 600 µm in fish length (seven sections spaced 100 µm) for each fish. Longitudinal images were acquired in an inverted Zeiss LSM 880 confocal microscope with 20X Plan-Apochromat dry objective. Images were acquired as *z*-stacks (six slices 1 µm apart).

To perform immunohistochemistry in wholemount SCs (electronic supplementary material, figure S2), samples were fixed and placed in Scale A2 solution (4 M urea; 10% glycerol; 0.1% Triton X-100 [66]) at 4°C for at least 2 days. The SCs were then treated with collagenase (2 mg ml^−1^ in PBS) for 25 min at RT, followed by a wash in PBSTx and incubated in 50 mM glycine in PBSTx for 10 min at RT. Samples were then incubated in 10% blocking solution (10% goat serum; 1% DMSO; 1% Triton X-100 in PBS) at 4°C for 2 days. The solution was then substituted with blocking solution with primary antibody and incubated at 4°C for 5 days. The SCs were then washed in PBS at RT in a roller for several hours and afterwards incubated with blocking solution with secondary antibody at 4°C for 5 days. Samples were then washed in PBS for several hours and transferred to Scale A2. Samples were stored at 4°C until cleared and were acquired in the lighsheet microscope as described in the clearing subsection.

### 4.6. Tissue clearing in juvenile and adult zebrafish and image acquisition

Samples for tissue clearing were fixed as for tissue sectioning. The SC in adults was isolated from the vertebral column using forceps, except for samples that were imaged with vertebrae. In these samples, the neural arches were removed. After washing 3 × 5 min with PBS, the samples were incubated with Scale A2 solution at 4°C, protected from light, until cleared (minimum of two weeks). For confocal imaging, samples were kept in Scale A2 and placed in a glass-bottom Petri dish (FluoroDish, #FD35COL, WPI) with a minimum of liquid. Samples were imaged in an inverted Zeiss LSM 880 confocal microscope with 25X LCI Plan-Neofluar Corr DIC water immersion objective. Images were acquired as *z*-stacks with slices 2.751 µm apart with the maximum depth possible (less than 100 µm). For light sheet imaging, samples were transferred to Scale S4 (40% D-(-)-sorbitol; 4 M urea; 10% glycerol; 0.2% Triton; 15% Dimethylsulfoxide [67]) the day before imaging and kept at 4°C protected from light. Samples were imaged in a Zeiss light sheet Z1 microscope with a 20X Clr Plan-Neofluar Corr nd = 1.45 85 mm clearing immersion objective. During imaging the SC was suspended in clearing solution from a glass capillary (size 1 / approximately 0.68 mm). Dual-side images of injured or control regions were acquired as *z*-stacks with slices 1.14 µm apart along the whole depth of the SC. Dual-side stacks were fused after acquisition using ZEN 2014 SP1 software.

### 4.7. Live imaging of blood flow in juvenile zebrafish

To visualize the flow of thrombocytes inside SC vessels *Tg(-6.0itga2b:eGFP)^la2^*; *Tg(kdrl:HRAS-mCherry)^s896^* fish with 3–4 wpf (with melanocytes) were mounted in 1% low-melt agarose (Nusieve GTG Agarose, #500800, Lonza) containing 0.375 mM tricaine on a glass-bottom dish with E3 medium (5 mM NaCl; 0.17 mM KCl; 0.33 mM CaCl2; 0.33 mM MgSO4) with 0.375 mM tricaine. A single plane was acquired at the level of the central canal of the SC in an inverted Zeiss LSM 880 confocal microscope with a 10x EC Plan-Neofluar dry objective, in a time-stack of three frames per second (fps).

To visualize blood flow at higher imaging speed in a light sheet microscope, *casper* fish were used, since melanocytes block the light sheet path. To fluorescently label blood, we injected a solution of 50 mg ml^−1^ of Dextran, Alexa Fluor 647 (10 000 MW, #D22914, Invitrogen) in water into the heart of 3–4 wpf *casper* fish. Fish were anaesthetized in 0.375 mM tricaine solution, placed on a sponge with the ventral side up and the dextran-A647 solution was injected into the heart using a glass needle attached to a suction tube and a mouthpiece. The tracer dye was allowed to circulate for 15–30 min and individual fish were placed in 1% low-melt agarose containing 0.375 mM tricaine and supported head down from a glass capillary (size 3/approx 1.5 mm) in an imaging chamber filled with E3 medium containing 0.375 mM tricaine. A single plane was imaged at the level of the central canal of the SC in a Zeiss light sheet Z1 microscope with a 20X W Plan-Apochromat DIC 75 mm water immersion objective, in a time-stack of 57fps.

### 4.8. Dextran Alexa Fluor 647 heart injection and detection

To evaluate vessel perfusion and permeability properties, we injected a 10 KDa dextran-A647 into circulation. Either *Tg(kdrl:HRAS-mCherry)^s896^* or *Tg(kdrl:NLS-mCherry)^is4^* lines were used for the injection. Fish were anaesthetized by immersion in 0.6 mM tricaine and placed with the ventral side up on a sponge soaked with tricaine solution. An incision was performed at the level of the heart. The heart was injected with 0.3–0.5 µl of dextran-A647 solution (50 mg ml^−1^ in water) in a glass needle, using a stereoscope to visually control the entry of the dye in the heart. For the short-term and long-term perfusions, fish were either kept in the sponge for 1 min or returned to system water for 30 min, respectively, before sacrificing the fish and collecting the vertebral column. SCs were fixed and imaged whole (1 min perfusion) or sectioned longitudinally (25 µm-thick) as described above (30 min perfusion). To mount the slides, gelatine was removed from cryosections using PBS heated to 37°C (4 × 5 min washes). The slides were then incubated with DAPI for 20 min at RT and mounted in Mowiol. Imaging of wholemount SCs was performed in a light sheet microscope as described above. Imaging of sections was performed in an inverted Zeiss LSM 880 confocal microscope with 20X Plan-Apochromat dry objective. Images were acquired as *z*-stacks (six slices 1 µm apart) taken at mid-section depth. The rostral and caudal sides of the injury and a region 3 mm caudal to the injury were imaged in three adjacent sections that included or were near the central canal.

### 4.9. Edu incorporation and detection

To label proliferating cells, we used the thymidine analogue EdU (5-ethynyl-2′-deoxyuridine) that is incorporated into DNA during active DNA synthesis. The EdU solution (2.5 mg ml^−1^ dissolved in PBS) was injected intraperitoneally (volume 20–40 µl adjusted to the size of the fish) using a 0.5 ml U-100 insulin syringe with 30G needle (no. 324825, BD Micro-Fine). EdU injections were performed in the time points described in the figure schematics. SC samples were collected, fixed and sectioned longitudinally (25 µm-thick) as described above. For EdU detection, gelatine was removed from cryosections using PBS heated to 37°C (4 × 5 min washes), followed by incubation with 3% blocking solution (3% bovine serum albumin (BSA); 0.5% Triton X-100 in PBS) for 30 min at RT. The Click-it reaction cocktail was prepared as described in the kit instructions (Click-iT EdU Cell Proliferation Kit for Imaging, Alexa Fluor 647 dye, #C10340, Invitrogen), but with half the concentration of Alexa-647 dye. The slides were covered with 150–200 µl of reaction solution and incubated for 30 min in the dark. After 3 × 5 min washes in 3% blocking solution, the slides were stained with DAPI, mounted and imaged as described for dextran-A647 detection.

### 4.10. Transmission electron microscopy

The vertebral column of adult zebrafish was dissected and the SC isolated from the vertebrae before fixation overnight at 4°C in 0.1 M sodium cacodylate buffer, pH 7.4, containing 2,5% (v/v) glutaraldehyde. Following 1 h post-fixation in 1% (aq.) osmium tetroxide and 30 min contrast in block in 1% (aq.) uranyl acetate. Dehydration was made using ethanol gradient (50–70–95–100%) and infiltration was aided using propylene oxide and a mixture (1 : 1) of propylene oxide and EPON 812 resin (EMS). Samples were embedded in EPON resin and hardened at 60°C for 36 h. Sections were obtained using an ultramicrotome Reichert Supernova (Leica microsystems), semi-thin sections (500 nm) were stained with toluidine blue for light microscope evaluation. Ultra-thin sections (70 nm) were collected in Formvar (AGAR scientific) coated copper slot grids, and counter-stained with uranyl acetate and lead citrate (Reynold recipe), and screened in a Hitachi H-7650 transmission electron microscope at 100 kV acceleration.

### 4.11. Quantitative PCR

For injury-induced qPCR analysis, total RNA was extracted from twenty pooled 4 mm SC fragments including the injury site. RNA extraction was performed using TRIzol and RNeasy Mini Kit (Qiagen no. 74104) according to the manufacturer's instructions. The purified RNAs were reverse transcribed with the iScriptTM cDNA Synthesis Kit (Bio-Rad) and qPCR was performed with Power SYBR Green PCR Master mix (Applied Biosystems) and a 7500 Fast Real-time PCR system (Applied Biosystems). All experiments were completed using biological replicates, expression levels were normalized to *gapdh* as control and the relative expression values were obtained using the ΔΔCT method. Primer sequences are noted in electronic supplementary material, table S3. Normalized values were plotted using ggplot2 and analysed statistically using GraphPad Prism 8.

### 4.12. Quantification and statistical analysis

Quantified data were organized and processed using Numbers spreadsheet (Apple) and RStudio (version 1.4.1717). Data plotting was performed in RStudio (ggplot2 package v3.3.5). Plotted data were presented as violin plots with quartiles or as mean ± standard deviation (SD). Statistical analysis was performed using GraphPad Prism 8, unless stated otherwise. Data were tested for normal distribution using Shapiro–Wilk normality test and parametric (One-way ANOVA followed by Dunnett's multiple comparisons test; Unpaired *t*-test) and non-parametric tests (Kruskal–Wallis test followed by Dunn's multiple comparisons *post hoc* test; Wilcoxon matched-pairs signed rank test; Mann–Whitney test) were used accordingly. All *p*-values were included in the graphs, with the exception of graphs with multiple comparison tests between all conditions, in which only *p*-values < 0.05 were shown.

#### 4.12.1. Quantification of number of vessels in juvenile fish

Images of transversal sections acquired with the DM5000b microscope were scaled and the fluorescent channels merged using Fiji. In each image, the SC was measured for area, dorsal-ventral and left-right lengths using Fiji. For each image, the SC limits were defined and blood vessels inside were counted manually.

#### 4.12.2. Quantification of the position of arteries and veins in juvenile fish

Time-lapse confocal or light sheet images of blood flow inside the SC (either based on thrombocyte movement or flow of A647-labelled blood) were analysed in Fiji to identify the vessels where blood enters (arteries) and leaves (veins) the SC and obtain their position relative to the position of the intervertebral space in the v6-v12 abdominal vertebral region [68,69]. The vessel positions were then normalized to the size of the corresponding vertebra and plotted along to v6-v12 vertebrae or as a single plot independently of the vertebra identity. These data were also used to determine how many arteries and/or veins were detected in each vertebra, the distance between each vessel and its closest rostral vessel of the same type (arterial or venous) and the size of each vertebra.

#### 4.12.3. Quantification of the position of arteries and veins in adult zebrafish spinal cords

Projections of confocal images of the SC and attached vertebral bodies were analysed in Fiji to determine vessel identity (based on morphology) and distribution relative to the position of the intervertebral space in the v6-v14 abdominal vertebral region. These data were analysed and plotted as described for juvenile fish.

#### 4.12.4. Quantification of the spinal cord dorso-ventral length and vascular area in adult zebrafish spinal cords

Projections of confocal images of the SC at the level of v9–v11 vertebrae were analysed in Fiji to measure the dorso-ventral length. To quantify the vascular area, a region of interest (ROI) of 581 × 389 µm was defined at each the vertebral levels, the *kdrl:HRAS-mCherry^+^* pixels were defined using a threshold and the vascular area was defined as the fraction of positive pixels relative to the total number of pixels in the ROI.

#### 4.12.5. Kugler analysis of vessels in wholemount spinal cords

Data analysis of light sheet images was performed in Fiji, using an adapted three-dimensional image analysis workflow developed by Kugler *et al*. (https://github.com/ElisabethKugler/ZFVascularQuantification, https://doi.org/10.5281/zenodo.3978278 [70]. Quantification of vessel branch length, position and tortuosity was done as described in [33], with the following changes to the macro: manual ROIs with 500 × 500 × [z size] µm were defined for each SC image using Fiji's polygon tool and the threshold range for image segmentation was tested and adapted to our data set.

To plot the heatmaps of vessel length per bin the middle xy position of each vessel branch was calculated and binned in 100 × 100 µm bins in a 500 × 500 µm region at the site of the injury. The sum vessel length in each bin was then calculated for each sample and plotted as a heatmap. A similar approach was used to bin vessels according to rostral–injury–caudal position (200–100–200 µm bins along the RC axis) and dorsal–middle–ventral position (200–100–200 µm bins along the DV axis). To quantify the central-lateral positions the middle *z* positions of branches were normalized to the total *z* size of each SC and binned according to lateral-central-lateral position (25–50–25% bins). The total vessel length in each bin for each sample and time-point was then normalized to the average vessel length in each bin in the uninjured condition to obtain the fold change and the average normalized values per region and time-point.

#### 4.12.6. Quantification of number of endothelial nuclei in light sheet images of wholemount spinal cords

The number and position of *kdrl:NLS-mCherry*-positive nuclei per SC was quantified manually using the Cell Counter plugin available in Fiji, from maximum projections of light sheet images of whole SCs (500 × 500 × [*z* size] µm). To obtain the fold change from 1 dpi, the number of endothelial nuclei for each sample was normalized to the average nuclei number in the 1 dpi condition. Heatmaps of nuclei number and position were created as described for total vessel length.

#### 4.12.7. Quantification of extravasated dextran-A647 in spinal cord sections

The intensity levels of dextran-A647 were quantified in six 50 × 50 × 5 µm regions of interest (ROIs) in maximum projection images, positioned in the extravascular tissue and excluding the central canal (electronic supplementary material, figure S5A-B). The average intensity between ROIs in each image was then averaged between the three histological sections for each sample.

#### 4.12.8. Quantification of proliferating ECs in spinal cord sections

The number of *kdrl:NLS-mCherry* positive and *kdrl:NLS-mCherry*/EdU double positive nuclei per ROI were quantified manually using the Cell Counter plugin available in Fiji, from maximum projection images of longitudinal sections (850.2 × 425.1 × 5 µm region centred in the injury). The proportion of proliferating ECs was calculated as the fraction of *kdrl:NLS-mCherry*/EdU double positive nuclei over the total number of *kdrl:NLS-mCherry* nuclei. The absolute or relative values for each ROI were then averaged between the three histological sections for each sample.

#### 4.12.9. Quantification of number of ECs and pericytes in confocal images of wholemount spinal cords

The number of *kdrl:NLS-mCherry* nuclei and *pdgfrb:citrine* cells was quantified manually in maximum projections of confocal images of the left region of cleared SCs (340.08 × 340.08 × 90 µm depth) using the Cell Counter plugin available in Fiji. The number of ECs was normalized to the number of pericytes to obtain the EC/pericyte ratio.

#### 4.12.10. Quantification of the fraction of vessels with no/low *gfap:GFP* coverage

The vessel area was quantified in a 212.55 × 212.55 µm region in the injury site using Fiji. The vessels were defined using a threshold and vessel area was quantified using the ‘Analyse Particles’ plugin. The vessels with no/low associated *gfap:GFP* signal were manually selected and the corresponding vessel area was measured and divided by the total vessel area in the region.

## Data Availability

The datasets supporting this article have been uploaded as part of the electronic supplementary material [71].
